# Efficacy and safety of chlorhexidine vs. povidone-iodine for vaginal antisepsis in surgery: an updated systematic review and meta-analysis

**DOI:** 10.1097/MS9.0000000000004311

**Published:** 2025-11-18

**Authors:** Areej Zeeshan Ahmed, Maasfah Tahir, Muhammad Suhaib Hanif, Hamzah Naushad Siddiqui, Sajal Irshad, Khadija Jalal, Jalib Ahmed, Simra Shadab, Yashfa Parveen, Saniha Outmani, Ummi Aiman Rahman, Wania Ahmer, Mohammad Aadil Qamar, Pratik Bhattarai, Omar Irfan, Zohra S. Lassi

**Affiliations:** a Department of Medicine, Ziauddin University, Karachi, Pakistan; b Department of Medicine, Dow University of Health and Sciences, Karachi, Pakistan; c Department of Medicine, Dow International Medical College, Karachi, Pakistan; d Department of Medicine, Liaquat National Hospital & Medical College, Karachi, Pakistan; e Department of Medicine, Akhtar Saeed Medical College, Rawalpindi, Pakistan; f Department of Medicine, King Edward Medical College, Lahore, Pakistan; g Department of Medicine, Manipal College of Medical Sciences, Pokhara, Nepal; h Department of Medicine, Aga Khan University Hospital, Karachi, Pakistan; i School of Public Health, Faculty of Health and Medical Sciences, University of Adelaide, Adelaide, Australia; j Robinson Research Institute, University of Adelaide, Adelaide, Australia

**Keywords:** chlorhexidine gluconate, gynecologic surgery, obstetric surgery, povidone-iodine, preoperative vaginal antisepsis, surgical site infections

## Abstract

**Background:**

Surgical site infections (SSIs) are significant postoperative complications in gynecologic and obstetric surgeries. Preoperative vaginal antisepsis is vital for prevention. This review compares the efficacy and safety of chlorhexidine gluconate (CHG) vs. povidone-iodine (PI) for vaginal antisepsis in preventing postoperative infections.

**Materials and Methods:**

A systematic search of major databases was conducted through 8 Ja-nuary 2025. Randomized controlled trials (RCTs) comparing CHG and PI for vaginal antisepsis during urogynecologic procedures were included. Meta-analyses were performed using random-effects models to estimate risk ratios (RRs) with 95% confidence intervals (CIs). The GRADE approach assessed the certainty of evidence. The primary outcome was SSIs; secondary outcomes included febrile morbidity, hospital stay, antiseptic-related side effects, and wound complications.

**Results:**

Twelve RCTs, including 4936 participants, were analyzed. The results showed that CHG significantly reduced compared to PI (RR: 1.71; 95% CI: 1.30–2.26; *I*^2^ = 17%; high-certainty evidence). Subgroup analyses showed higher SSI risk with PI in upper/lower-middle-income countries (RR: 1.79; 95% CI: 1.17–2.74) and high-income countries (RR: 1.66; 95% CI: 1.08–2.56). PI was also associated with increased risks of superficial SSIs (RR: 1.57), deep SSIs (RR: 2.35), and readmissions (RR: 1.59).

**Conclusion:**

CHG was superior to PI in preventing SSIs in gynecologic and obstetric surgeries. With high-quality evidence supporting its use, CHG is recommended for vaginal preparation. Future research should explore cost-effectiveness and long-term outcomes.

**HIGHLIGHTS**

- Compared to povidone-iodine (PI), chlorhexidine gluconate dramatically decreased surgical site infections (SSIs).
- 4936 women undergoing urogynecologic surgery were included in a meta-analysis of 12 randomized controlled trials.
- Chlorhexidine outperformed all SSI types, including deep and superficial infections.
- Chlorhexidine is the recommended vaginal antiseptic, according to high-certainty evidence; PI was linked to increased readmission rates following surgery.

## Introduction

Surgical site infections (SSIs) that occur in surgical wounds are a major cause of healthcare-associated infections (HCAIs)^[1]^. They are the most common and costly postoperative complications and are the reason for morbidity and prolonged hospitalizations^[2]^. SSIs pose a significant risk in obstetric and gynecological surgeries, as vaginal microorganisms may contaminate incision sites, increasing costs and morbidity^[3]^. Vaginal antisepsis is essential to minimize the risk of gynecologic infections and improve patient outcomes^[4]^.

Globally, povidone-iodine (PI) is one of the most widely used antiseptics due to its cost-effectiveness and bactericidal properties^[5]^. PI releases free iodine in solutions that penetrate microbial cell walls to destroy bacterial proteins and DNA^[6]^. Chlorhexidine gluconate (CHG) is another widely used antiseptic, with bactericidal properties against gram-negative and gram-positive bacteria, and it works by disrupting bacterial cell membranes^[7]^. The Federal Drug Authority (FDA) has approved PI for vaginal preparation, and the American College of Obstetricians and Gynecologists (ACOG) states that it is safe to use up to 4% CHG for vaginal antisepsis. However, the use of CHG for vaginal antisepsis is off-label, and extensive research on its safety and efficacy is needed^[8,9]^.

Existing literature supports the efficacy and safety of both agents across various clinical contexts^[8,9]^. Currently, there is no recommendation for a specific skin antiseptic. Studies have shown that chlorhexidine-alcohol solutions may have greater efficacy, easier application, improved durability, and a superior cost profile compared to PI^[10–12]^. However, the evidence remains insufficient. Additionally, research indicates that alcohol-based antiseptics should not be applied to mucous membranes, which limits their use in certain surgical procedures^[10–12]^.

This study stems from the considerable impact SSIs have on patient outcomes and healthcare systems. Despite the widespread use of these antiseptic agents, the lack of conclusive comparative data highlights a critical gap in our understanding. This paper aims to delve into the impact of these antiseptic agents in obstetric and gynecological surgeries, aiming to provide clear and definitive recommendations for clinical practice.

## Methods

The protocol for this study was registered with PROSPERO (Registration ID: CRD42024566304), and no deviations were made from the registered protocol during the conduct of this review. This systematic review is reported in accordance with the Preferred Reporting Items for Systematic Reviews and Meta-analysis (PRISMA)^[13]^. The PRISMA checklist can be accessed in PRISMA checklist. The review also adheres to the Transparency In interventional systematic reviews and Network meta-analyses (TITAN) checklist to enhance transparency and completeness of reporting; the completed TITAN checklist is available in the Supplemental Digital Content 1, available at: http://links.lww.com/MS9/B23^[14]^. The AMSTAR grading of this review is “low” and can be accessed in the AMSTAR Checklist. This rating was determined using the AMSTAR 2 tool for evaluating the methodological quality of systematic reviews of randomized trials.

The Patients, Interventions, Comparisons, and Outcomes (PICO) for this study consisted of adult women (≥18 years old) who were part of a randomized controlled trial (RCT) undergoing gynecological/obstetric procedures that compared CHG to PI for vaginal antisepsis, with SSI as the primary outcome of interest. RCTs were selected exclusively due to their ability to minimize bias and provide the highest level of evidence for intervention efficacy. Only studies published in English were considered for inclusion.

### Search strategy

A comprehensive search of electronic databases, including PubMed, Google Scholar, Cochrane Library, and China National Knowledge Infrastructure, was executed to identify relevant studies available from inception (January 1985) until 8 January 2025. Our search strategy employed a combination of subject headings and keywords to ensure we were not missing any relevant studies. Moreover, subject experts were consulted to ensure a comprehensive search strategy. The included terms were related to study designs, such as “randomized controlled trial”; interventions like “chlorhexidine” and “povidone-iodine”; and outcomes, specifically “surgical site infection.” The detailed electronic search terms and strategies used are found in Supplemental Digital Content Table S1, available at: http://links.lww.com/MS9/B23. By using this approach, we aimed to ensure a thorough collection of literature for our analysis.

### Selection criteria

The total records retrieved by the search strategy were de-duplicated using Endnote^[15]^. Following de-duplication, the records were exported to Microsoft Excel for screening. Two reviewers independently screened titles and abstracts to identify potentially relevant articles. They retrieved these articles in full text for further examination to determine their eligibility for inclusion in the review, as discussed earlier in PICO.

The reference lists of the included RCTs were also cross-referenced to identify any additional articles that might meet the inclusion criteria. Although grey literature was not included in the final analysis, we performed a preliminary scan of grey sources such as conference abstracts and trial registries to ensure no major studies were missed from the review to maintain the focus on peer-reviewed and published research. This rigorous selection process ensured that only the most relevant and high-quality studies were included in the final analysis.

Following this, the resulting full-text articles were independently reviewed by two authors, who documented the reasons for any exclusions to maintain transparency in the selection process. Any discrepancies and inconsistencies during the screening process were resolved by mutual discussion or by involving a third senior author.

Efforts were made to retrieve missing data where applicable. Duplicate studies were carefully identified and excluded from the review, while multiple reports of the same study were combined to ensure that each unique study served as the primary unit of analysis.

### Data collection

Data collection and analysis were conducted in accordance with the Cochrane Handbook for Systematic Reviews of Interventions^[16]^.

Data extraction was performed independently by two reviewers using standardized data extraction sheets in Microsoft Excel. Discrepancies were resolved through discussion or, if necessary, by consulting a third reviewer.

### Risk of bias assessment

In the included RCTs, the risk of bias was assessed by the Cochrane risk-of-bias tool for randomized trials (RoB 2)^[17]^ and applied to all the included studies in the meta-analysis.

Two independent reviewers performed the quality assessment, and disagreements from the reviewers were referred for consensus agreement by a third reviewer, making the evaluation stringent and objective.

### Data synthesis

Meta-analysis was conducted when three or more studies reported the same outcome, following Cochrane Collaboration guidelines.

Pooled effect estimates were calculated using mean difference (MD) with 95% confidence intervals (CIs) for continuous outcomes, and risk ratio (RR) with 95% CIs for dichotomous outcomes. A random-effects model was applied using Review Manager (RevMan)^[18]^.

Heterogeneity was assessed using Higgins’ *I*^2^ statistic. An *I*^2^ value greater than 50% was considered indicative of significant heterogeneity. In such cases, a leave-one-out sensitivity analysis was performed to identify the studies contributing most to heterogeneity.

For studies that reported continuous outcomes as medians with interquartile ranges or minimum and maximum values, the mean and standard deviation were estimated using the online Mean and Variance Estimation tool (https://www.math.hkbu.edu.hk/~tongt/papers/median2mean.html), assuming a normal distribution. Skewness was assessed using methods described by Shi *et al* (2023)^[19]^. If data were found to be significantly skewed, the respective study was excluded from the analysis.

A funnel plot was also generated using RevMan to assess potential publication bias in analyses containing 10 or more studies. In case of asymmetry or suspicion of bias, we conducted a sensitivity analysis by sequentially excluding individual studies (leave-one-out method) to identify those contributing most to the asymmetry.

In the study by Marinone *et al*^[20]^, the duration of hospital stay was reported as the number of participants associated with specific duration values (e.g., 1 day = number of participants/total). To convert this categorical data into means and standard deviations, we used an online Standard Deviation Calculator (https://www.calculator.net/standard-deviation-calculator.html).

The study by Gezer *et al*^[21]^ presented subgroup data for CHG and PI based on antiseptic temperature (cold: 25°C and warm: 37°C). These subgroups were combined for each antiseptic using the online calculator from StatsToDo (https://www.statstodo.com/CombineMeansSDs.php). This method follows guidelines outlined in the Cochrane Handbook for Systematic Reviews of Interventions version 6.0^[16]^ and the textbook *Statistics with Confidence* by Altman *et al*^[22]^.

Subgroup analyses were also conducted based on the depth of the wound (superficial vs. deep) and the income classification of countries, as defined by the World Bank^[23]^.

### GRADE analysis

The certainty of evidence was assessed using Grading of Recommendations, Assessment, Development, and Evaluation (GRADE) criteria^[24]^. GRADE evidence profiles were developed for each outcome to evaluate five key domains: risk of bias within studies, directness of evidence, consistency (heterogeneity), precision of effect estimates, and potential publication bias. Based on these domains, each primary outcome was rated as “high,” “moderate,” “low,” or “very low.”

## Results

### Selection process

**Figure 1.**
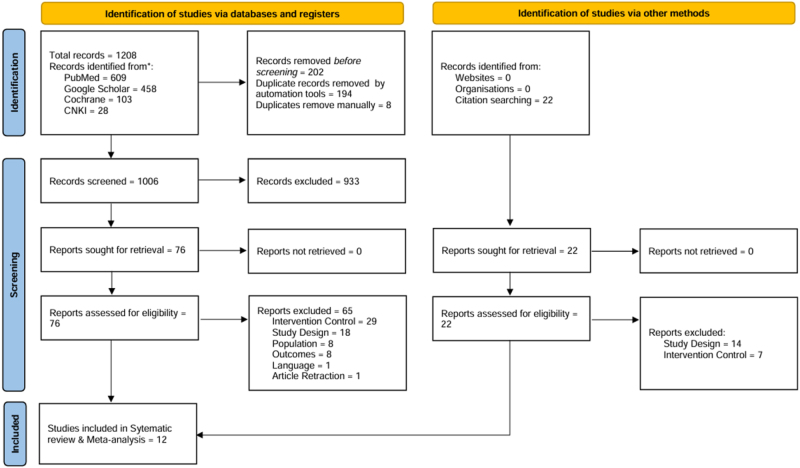
PRISMA flow diagram.

The electronic literature search yielded a total of 1208 records from a variety of databases that underwent title and abstract screening. Records were removed manually and excluded based on irrelevance, differing populations, outcomes, language, intervention and control groups, study design, and animal studies. Seventy-six original, and 22 articles identified via reference searching underwent full-text screening using predefined criteria, where 89 studies were excluded. The reference lists of the reviews retrieved were also screened for additional articles, but no other relevant articles were identified responding to our inclusion criteria. Twelve RCTs were finally included in this review^[20,21,25–34]^. A PRISMA flow diagram for our search methodology and results is presented in Figure 1.

### Study characteristics

Twelve RCTs published between 2005 and 2023 were included, involving a total of 4936 participants. Participants received either chlorhexidine-alcohol (CHG-alcohol) or PI as a preoperative intervention. The mean age ranged from 22.6 to 62.2 years. The primary objective of the included studies was to assess postoperative SSI.

Superficial SSI was reported as an outcome by seven studies^[21,25,28–30,33,34]^, whereas deep SSI was reported by five studies^[25,28,30,33,34]^.

Four studies^[20,21,26,27]^ used 4% chlorhexidine and 10% PI in isopropyl alcohol for preoperative antisepsis before procedures such as hysterectomy, elective cesarean section, and surgical resections for malignant or premalignant lesions. Among these, two studies reported identifiable organism growth in addition to common SSI.

Five studies^[28–30,32,33]^ employed 2% CHG in isopropyl alcohol as the intervention, with 10% PI serving as the control, specifically before cesarean sections and uro-gynecological procedures. Two additional studies^[24,33]^ used 2% CHG and 7.5% PI, while one study^[31]^ included 4% CHG and 7.5% PI. Furthermore, a recent study^[20]^ conducted in 2023 evaluated the efficacy of two intervention groups, 2 and 4% CHG, and 10% PI for vaginal antisepsis.

All included trials were also categorized based on the World Bank classification of economies, with eight studies being labelled as High-Income Countries and four being labelled as Upper/Lower Middle-Income Countries.

Table 1 summarizes data from 12 RCTs, encompassing 4936 participants, included in our analysis. The table presents the baseline characteristics of the included studies, encompassing a range of detailed information. This includes the names of the authors, the year of publication, and the specific gynecological procedures examined. Additionally, it provides the percentage distribution of participants in the intervention and control groups, along with the total number of participants involved in each study. The table also reports the mean age of the participants, offering a comprehensive overview of the demographic and methodological aspects of the studies.

**Table 1:** Characteristics of studies included in the analysis

		Concentration of antiseptic (%)		Number of participants		Mean age± standard deviations				
First author	Surgical procedure	CHG	PI	CHG	PI	CHG	PI	Country	Time frame	Funding source
Culligan *et al* 2005^a^	Vaginal hysterectomy with or without reconstructive pelvic	4	10	23	27	42.6 ± 7.8	45.0 ± 11.5	USA	October 2002 and September 2003	No outside funding source
Springel *et al* 2017^a^	Cesarean section	2	7.5	461	471	28.4 ± 6.7	28.0 ± 6.0	USA	March 2014 and June 2016	No funding
Salama *et al* 2016^b^	Elective and nonelective cesarean	2	10	196	194	26.7 ± 4.6	26.6 ± 4.6	Egypt	June 2014 to December 2014	Not mentioned
Gezer *et al* 2019^a,b^	Surgery for malignant/premalignant	4	10	109	110	53.2 ± 12.7	53.0 ± 12.0	Turkey	March 2014 and June 2016	No funding
Hill *et al* 2019	Total hysterectomy	4	10	44	41	57.6 ± 10.9	62.2 ± 11.6	USA	May 2018 and August 2019	Funding was provided by the TriHealth Medical Education Research Fund
Kesani *et al* 2019^a,b^	Cesarean section	2	10	273	287	22.6 ± 2.7	22.6 ± 2.6	India	April 2017 to September 2017	No funding
Marinone *et al* 2023^a^	Laparoscopic or robotic hysterectomy	3	10	34	16	58.8 ± 13.6	61.4 ± 13.5	USA	1 February 2020 to 31 March 2021	Fred and Irmi Bering Endowed Chair for Laparoscopic Surgery Fund at Danbury Hospital
Lakhi *et al* 2019	Elective cesarean	2	10	524	590	32.5 ± 5.6	32.6 ± 5.2	USA	1 December 2016 through 28 February 2018	Not mentioned
Albezrah *et al* 2019	Cesarean section	2	7.5	490	493	29.7 ± 3.5	29.5 ± 3.3	Saudia Arabia	January 2018, to February 2019	Not mentioned
Luwang *et al* 2021^b^	Elective/emergency cesarean section	2	10	149	151	28.2 ± 4.8	27.9 ± 4.2	India	July 2016 to October 2017	No funding
Rockefeller *et al*	Uro-gynecologic procedures	2	10	61	58	58.0 ± 13.0	57.0 ± 12.0	USA	August 2019 and January 2021	No funding
Rastogi *et al* 2020	Diagnostic/operative hysteroscopy	4	7.5	68	66	44.0 ± 9.0	46.0 ± 10.0	USA	9 October 2017	This work was supported by the Albert B. Gerbie Professorship fund

CHG, chlorhexidine-gluconate; PI, povidone-iodine.

^a^ These studies did not report age in the format of mean and standard deviation; hence, stated values have been calculated using an online calculator.

^b^ Countries categorized as upper/lower middle-income countries.

The proportion of participants treated with CHG vs. PI varied across studies, ranging from 2 to 4% CHG and 10% PI. The mean age of participants differs significantly, with older populations studied in Hill *et al*^[27]^ and Rockefeller *et al*^[32]^, while younger cohorts are included in studies like Kesani *et al*^[28]^ and Lakhi *et al*^[29]^.

### Quality assessment

**Figure 2.**
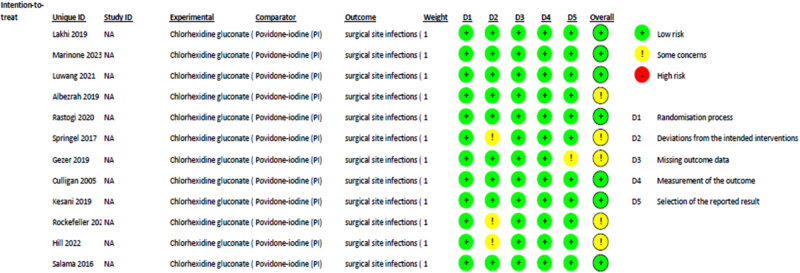
Quality assessment of the included RCTs using RoB 2.0.

Among the twelve studies included, seven had a low risk and five had some concern for bias (Fig. 2). The most common reasons for bias were deviations from intended interventions, measurement of the outcome, and selection of the reported result. Sensitivity analysis was conducted appropriately to evaluate the effect of the exclusion of studies with a risk of bias.

A summary of the data analysis, including calculated effect sizes for all the outcomes and measures of heterogeneity, is presented in Table 2.

**Table 2:** Summary of outcomes and data analysis

	Outcomes	Number of studies	Meta-analysis		Measure of heterogeneity(*I*^2^)		Certainty of the evidence (GRADE)
			Measure of relative effect (RR/MD) with 95% CI	*P*-value	*I*^2^ overall (%)	*I*^2^ (%) after sensitivity analysis	
Efficacy outcomes	Postoperative SSIs	12	1.71 (1.30, 2.26)	*P* = 0.0001	17		High
	Superficial SSIs	7	1.57 (1.18, 2.08)	*P* = 0.002	0		High
	Deep SSIs	5	2.35 (1.24, 4.45)	*P* = 0.009	0		High
	Hospital readmission rates	5	1.59 (1.01, 2.51)	*P* = 0.05	24		High
	Duration of hospital stay	5	0.08 (−0.57, 0.72)	*P* = 0.82	92	48	Low
	Growth of *E. coli*	3	1.62 (0.82, 3.23)	*P* = 0.17	0		Moderate
	Growth of *A. baumannii*	3	1.18 (0.30, 4.59)	*P* = 0.81	0		Low
	Growth of *Klebsiella*	3	1.53 (0.27, 8.75)	*P* = 0.63	0		Low
	Growth of *S. epidermis*	3	1.31 (0.39, 4.38)	*P* = 0.66	33		Moderate
Safety outcomes	Postoperative wound infection	3	1.96 (0.85, 4.49)	*P* = 0.11	59	0	Moderate
	Postoperative wound seroma or hematoma	6	1.09 (0.72, 1.65)	*P* = 0.69	0		Moderate
	Postoperative wound dehiscence	4	1.27 (0.68, 2.40)	*P* = 0.45	43		Low
	Postoperative endometritis	4	1.18 (0.63, 2.22)	*P* = 0.60	0		Moderate

CI, confidence interval; MD, mean difference; RR, risk ratio; SSIs, surgical site infections.

For all outcomes except 3 (postop wound dehiscence, cultures positive for *Escherichia coli* and cultures positive for *Staphylococcus epidermis*), the Relative Effect was measured as PI vs. CHG.

The Summary of Findings table is reported as Supplemental Digital Content Table S2, available at: http://links.lww.com/MS9/B23. Out of the 17 outcomes assessed, 7 outcomes (41.2%) were reported as “High certainty of the evidence,” 6 outcomes (35.3%) were reported as “Moderate certainty of the evidence,” and 4 outcomes (23.5 %) were reported as “Low certainty of the evidence.” Low evidence was due to bias and imprecision within the study.

## Meta-analysis

### Postoperative Surgical Site Infections (SSIs)

**Figure 3.**
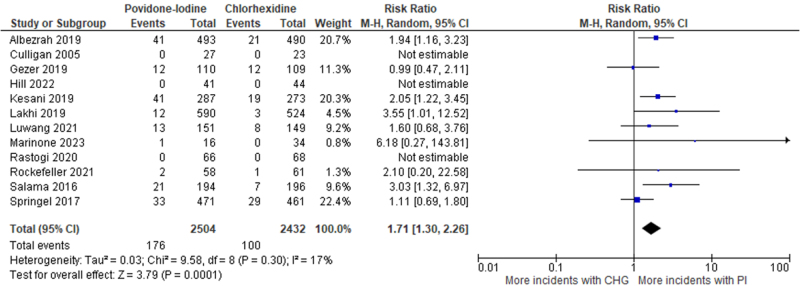
Forest plot of meta-analysis comparing PI to CHG with postoperative SSI as the outcome.

All twelve included RCTs (*n* = 4936 participants) reported postoperative SSIs as an outcome. The pooled analysis demonstrated that there was a statistically significant (*P*-value = 0.0001) higher incidence of SSIs with PI, as compared to CHG [RR: 1.71 (95% CI: 1.30–2.26), *I*^2^ = 17%, *n* = 12; GRADE: High certainty] (Fig. 3).

**Figure 4.**
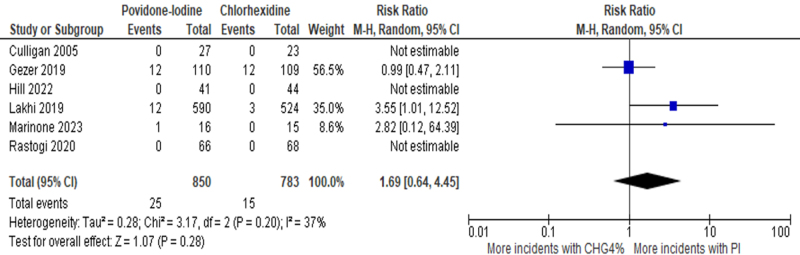
Forest plot of meta-analysis compares PI to 4% CHG with postoperative SSI as the outcome.

A second meta-analysis was conducted, only including studies that compared 4% CHG with PI. This included 6 studies (1633 patients), three of which reported zero events in both CHG and PI groups. Meta-analysis of the three remaining trials (1364 patients) showed that there was a nonsignificant higher risk of SSIs in the PI group [RR: 1.69 (95% CI: 0.64–4.45), *P* = 0.28, *I*^2^ = 37%, Moderate certainty of the evidence] (Fig. 4).

**Figure 5.**
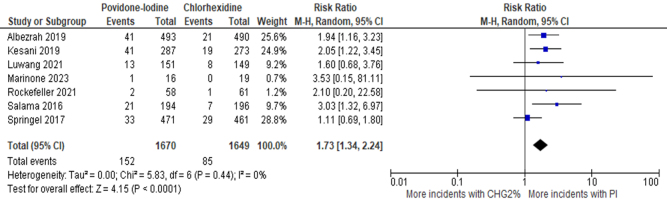
Forest plot of meta-analysis comparing PI to 2% CHG with postoperative SSI as the outcome.

Another meta-analysis was done, including only studies that compared 2% CHG to PI (7 studies, 3319 patients). The result revealed that the risk of SSIs was again higher with PI but was now statistically significant [RR: 1.73 (95% CI: 1.34–2.24), *P* < 0.0001, *I*^2^ = 0%, High certainty of evidence], favoring 2% CHG (Fig. 5).

### Superficial and deep Surgical Site Infections (SSIs)

Seven trials reported superficial SSIs as an outcome. Five of these reported a higher incidence of superficial infections with PI but the result was only significant in one study (Kesani *et al*^[28]^). Two studies (Gezer *et al*^[21]^ and Lakhi *et al*^[29]^) reported a higher incidence of CHG, but neither result was significant.

**Figure 6.**
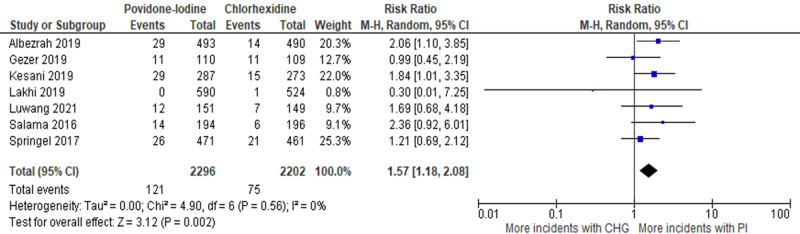
Forest plot of meta-analysis comparing PI to CHG with superficial SSI as the outcome.

Meta-analysis of these 7 trials (4498 patients) shows that the risk of superficial SSIs was significantly higher (*P* = 0.002) in the PI group [RR: 1.57 (95% CI: 1.18–2.08), *I*^2^ = 0%, High certainty of the evidence] (Fig. 6).

Five trials reported deep SSIs as an outcome. All but one study (Luwang *et al*^[30]^) reported a higher incidence of PI, and no studies reported a statistically significant result.

**Figure 7.**
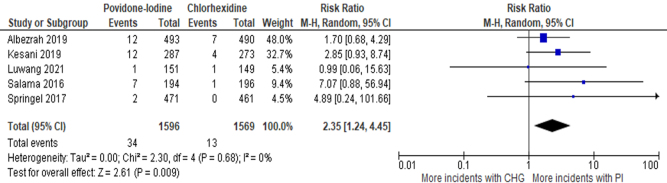
Forest plot of meta-analysis comparing PI to CHG with deep SSI as the outcome.

Meta-analysis of these 5 trials (3165 patients) showed that the risk of deep SSIs is significantly higher (*P* = 0.009) in the PI group [RR: 2.35 (95% CI: 1.24–4.45), *I*^2^ = 0%, High certainty of the evidence] (Fig. 7).

### Postoperative wound complications

Meta-analyses were done for postoperative wound complications, including wound infection, seroma or hematoma, and wound dehiscence.

#### Postoperative wound infection

Meta-analysis of the 3 studies (2436 patients) reporting postoperative wound infection revealed a nonsignificant higher risk of postoperative wound infection with PI [RR: 1.96 (95% CI: 0.85–4.49), *P* = 0.11, *I*^2^ = 59%, Moderate certainty of the evidence] (Supplemental Digital Content Figure S1, available at: http://links.lww.com/MS9/B23).

#### Postoperative wound seroma or hematoma

Meta-analysis of the 6 studies (2659 patients) reporting wound seroma or hematoma as an outcome revealed a nonsignificant higher risk of wound seroma or hematoma with PI [RR: 1.09 (95% CI: 0.72–1.65) *P* = 0.69, *I*^2^ = 0%, Moderate certainty of the evidence] (Supplemental Digital Content Figure S2, available at: http://links.lww.com/MS9/B23).

#### Postoperative wound dehiscence

Meta-analysis of the 4 studies (2184 patients) reporting wound dehiscence as an outcome revealed that there is a nonsignificant higher risk in the CHG group [RR: 1.27 (95% CI: 0.68–2.40), *P*=0.45, *I*^2^=43%, Low certainty of the evidence] (Supplemental Digital Content Figure S3, available at: http://links.lww.com/MS9/B23).

### Postoperative endometritis

Meta-analysis of the 4 studies (3419 patients) documenting postoperative endometritis revealed a nonsignificant higher risk in the PI group [RR: 1.18 (95% CI: 0.63–2.22), *P* = 0.60, *I*^2^ = 0%, Moderate certainty of the evidence] (Supplemental Digital Content Figure S4, available at: http://links.lww.com/MS9/B23).

### Hospital readmission rates

Five trials (3638 patients) reported the rate of readmission after surgery. All but one (Lakhi *et al*^[29]^) reported a higher incidence of readmission with PI, but the result was only significant in Salama *et al*^[33]^.

Meta-analysis showed that the incidence of readmission was significantly higher in the PI group [RR: 1.59 (95% CI: 1.01–2.51), *P*: 0.05, *I*^2^: 24%, High certainty of the evidence] (Supplemental Digital Content Figure S5, available at: http://links.lww.com/MS9/B23).

### Duration of hospital stay

Six studies (2453 patients) compared the duration of hospital stay after surgery with both antiseptics as an outcome. Hill *et al*^[26]^ reported data in the format: median (minimum and maximum). When attempting to calculate mean and standard deviation using methods outlined earlier, we found that the data for the CHG group were significantly skewed from normal distribution and the calculations would be unreliable. As such, Hill *et al*^[27]^ was purposely excluded from this analysis.

Meta-analysis of the 4 included studies (1552 patients) revealed a nonsignificantly higher hospital stay duration among patients in the PI group [MD: 0.08 (95% CI: 0.57–0.72), *P* = 0.82, *I*^2^ = 92%, Low certainty of the evidence] (Supplemental Digital Content Figure S6, available at: http://links.lww.com/MS9/B23).

### Growth of specific organisms in culture

Some studies have reported the organisms identified on culture from patients who developed SSIs. If three or more studies reported the growth of a specific organism, analysis was performed to compare the risk of growth between PI and CHG groups.

Meta-analyses were done, including three studies documenting the growth of *E. coli, Klebsiella*, and *Acinetobacter baumannii* on culture, separately for each organism.

For *E. coli*, a meta-analysis of 3 studies (579 patients) showed a nonsignificant higher risk of growth in the CHG group [RR: 1.62 (95% CI: 0.82–3.23), *P* = 0.17, *I*^2^ = 0%, Moderate certainty of the evidence] (Supplemental Digital Content Figure S7, available at: http://links.lww.com/MS9/B23).

The risk of growth was nonsignificantly higher in the PI group for both *A. baumannii* [3 studies, 579 patients, RR: 1.18 (95% CI: 0.30–4.59), *P* = 0.81, *I*^2^ = 0%, Low certainty of the evidence] (Supplemental Digital Content Figure S8, available at: http://links.lww.com/MS9/B23) and *Klebsiella* [3 studies, 579 patients, RR: 1.53 (95% CI: 0.27–8.75), *P* = 0.63, *I*^2^ = 0%, Low certainty of the evidence] (Supplemental Digital Content Figure S9, available at: http://links.lww.com/MS9/B23).

Three studies (669 patients) compared the growth of *S.epidermidis* and meta-analysis shows that there is a nonsignificant higher risk of growth in the CHG group [RR: 1.31 (95% CI: 0.39–4.38), *P* = 0.66, *I*^2^ = 33%, Moderate certainty of the evidence] (Supplemental Digital Content Figure S10, available at: http://links.lww.com/MS9/B23).

### Subgroup analysis for SSIs based on income status

**Figure 8.**
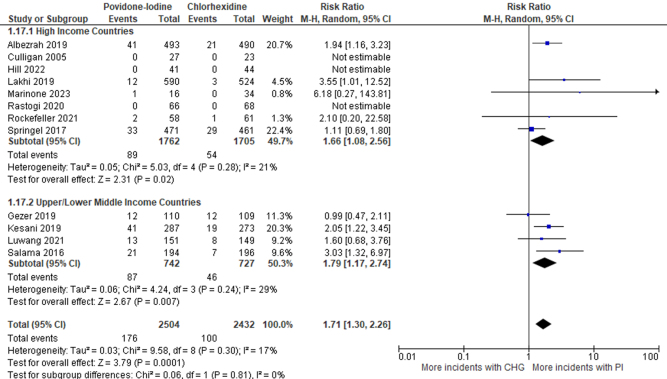
Forest plot of meta-analysis comparing PI to CHG with postoperative SSI as the outcome and studies divided into subgroups based on the income status of the country the trial was conducted in.

Eight studies (3467 patients) were classified into high-income countries [RR: 1.66 (95% CI: 1.08–2.56), *P* = 0.02, *I*^2^ = 21%, High certainty of the evidence], and 4 studies (1469 patients) in the Upper/Lower Middle-Income Countries subgroup [RR: 1.79 (95% CI: 1.17–2.74), *P* = 0.007, *I*^2^ = 29%, High certainty of the evidence]. In both subgroups, the risk of SSIs was significantly higher with PI than with CHG (Fig. 8).

### Sensitivity analysis

Sensitivity analysis was performed wherever the results for an outcome showed significant heterogeneity (*I*^2^ > 50%).

### Postoperative wound infection

For postoperative wound infection, sensitivity analysis was performed as there was significant heterogeneity in results (*I*^2^ = 59%). Removal of Springel *et al*^[34]^ caused the *I*^2^ value to decrease to 0%, revealing this study as the major source of heterogeneity. The exclusion of Springel *et al*^[34]^ made the result statistically significant [RR 3.25 (95% CI: 1.41–7.49), *P* = 0.006] (Supplemental Digital Content Figure S11, available at: http://links.lww.com/MS9/B23).

### Duration of hospital stay

For the duration of the hospital stay, sensitivity analysis was performed since there was significant heterogeneity (*I*^2^ = 92%). The exclusion of Albezrah *et al*^[25]^ reduced the *I*^2^ value to 48%, suggesting this study was the major source. After excluding this study, the results revealed that the duration of hospital stay was nonsignificantly higher in the chlorhexidine group [MD = −0.16 (95% CI: 0.55–0.23), *P* = 0.43] (Supplemental Digital Content Figure S12, available at: http://links.lww.com/MS9/B23).

### Publication bias

Publication bias was assessed for Figures 3 and 8. Funnel plots were generated for both outcomes to assess for asymmetry. These funnel plots are available in Supplemental Digital Content Figures S13 and S14, available at: http://links.lww.com/MS9/B23.

Analysis revealed noticeable asymmetry in both plots, suggesting potential publication bias. To further investigate this, a leave-one-out sensitivity analysis was conducted by sequentially excluding individual studies to identify those contributing most to the asymmetry. This approach identified Lakhi *et al* (2019), Marinone *et al* (2023), and Rockefeller *et al* (2021) as the primary sources of potential publication bias in our review.

## Discussion

To our knowledge, this meta-analysis is the first to systematically compare the efficacy and safety of CHG with PI in preventing SSIs in women undergoing preoperative vaginal antisepsis. For the overall SSIs, the meta-analysis shows a significantly higher risk associated with PI compared to CHG. These findings are consistent with those of Chen *et al*^[12]^ and Lee *et al*^[35]^. SSI typically occurs due to contamination of the wound by microorganisms. The source of these infectious agents can either be endogenous, originating from a patient’s own body, or exogenous, being airborne, originating from an operation theatre environment ^[19]^. CHG is a positively charged biguanide that binds to a negatively charged bacterial cell wall and disrupts its osmotic balance. At higher concentrations, it causes cytoplasmic contents to precipitate, which leads to cell death. With its wide range of microbicidal activity, it rapidly reduces resident skin flora as well as transient microorganisms. CHG’s longer duration of action prevents the regrowth of bacteria, thus prolonging the antiseptic effect^[21]^. On the other hand, PI is an iodine-releasing agent and is a complex iodine with polyvinylpyrrolidone. Free iodine acts as a bactericidal agent, destroying bacterial DNA and protein by oxidizing essential proteins, nucleotides, and fatty acids, eventually resulting in cell death^[6,22]^. PI is the standard and widely used antiseptic in clinical practice^[10]^.

The superiority of CHG can be attributed to the fact that it has equal bactericidal activity against both resistant and nonresistant bacteria, and it has better inhibitory and disinfectant effects than PI^[10]^. This is evident in different types of SSIs, including superficial SSIs and deep SSIs, where PI consistently underperformed relative to CHG. Similarly, Darouiche *et al*^[11]^ and Wang *et al*^[10]^ reported that CHG-alcohol was significantly more protective in reducing superficial and deep SSIs. This can be due to, as stated by Larson *et al*^[36]^, that PI becomes less efficacious in its antimicrobial activity in the presence of blood, whereas CHG remains effective even in the presence of body fluids. Also, CHG in an alcoholic medium dries faster when applied, reducing the waiting time for surgery^[12]^. The overall trend in the literature supports the conclusion that CHG, with its broader antimicrobial activity, is more effective in preventing postoperative infections compared to PI.

Another outcome of interest was postoperative wound infection, which did not yield statistically significant results initially, prompting a sensitivity analysis. Post-sensitivity analysis revealed that CHG demonstrated a statistically significant reduction in infection risk compared to PI. The significant outcome after sensitivity analysis can be due to CHG’s consistent antimicrobial properties, which remained effective across various study conditions and populations. This was also stated in the review conducted by Edward *et al*^[37]^, where more patients in the PI group developed postoperative wound infections.

Previous meta-analyses and systematic reviews, such as that by Lee *et al*^[35]^, did not specifically address endometritis and wound dehiscence when evaluating preoperative skin antiseptics like CHG and PI. However, recent RCTs provide valuable insights into these outcomes. Four RCTs – Lakhi *et al*^[29]^, Springel *et al*^[34]^, Albezrah *et al*^[25]^, and Salama *et al*^[33]^ – suggested a higher risk of endometritis with PI, though this was not statistically significant. Similarly, studies by Gezer *et al*^[21]^ and Springel *et al*^[34]^ noted a higher risk of wound dehiscence with CHG, but these findings also lacked statistical significance. Similarly, Li *et al*^[38]^ research underscored the connection between SSIs and longer hospital stays, which contributed to higher healthcare costs. Despite these significant clinical implications, these studies did not provide specific data on the comparative efficacy of PI and CHG in reducing the length of hospital stays. Cai *et al*^[7]^, however, supported the efficacy of CHG in reducing hospital stays without increasing adverse effects, such as deep vein thrombosis (DVT) or pulmonary embolism (PE). Despite the absence of systematic reviews focusing on hospital readmission rates after the use of these antiseptics, Salama *et al*^[33]^ reported a higher rate of sepsis-related readmissions with PI (10.3 vs. 2.6%, *P* = 0.002).

Although previous meta-analyses (e.g., Privitera *et al*^[16]^ and Lee *et al*^[12]^) have evaluated skin antiseptics broadly, they did not focus on gynecologic-specific outcomes like endometritis or wound dehiscence. These are especially relevant for cesarean deliveries and hysterectomies, where the surgical field involves areas with higher microbial colonization. Differences in infection rates may have a greater clinical impact in cesarean sections, while wound dehiscence remains a concern for open abdominal hysterectomies and emergency cesareans, particularly in patients with obesity or diabetes. Although our data did not show statistically significant differences, these findings highlight the need for procedure-specific antiseptic protocols tailored to patient risk factors.

Reviews, including Lee *et al*^[35]^, have assessed the effectiveness of CHG over PI in reducing bacterial load on the skin and preventing SSIs. In contrast to this, another study by Davies *et al*^[38]^ shows that complete decolonization rates were greatest with a combination of CHG and PI (90%). However, recent studies have not consistently favored one antiseptic over the other. Some studies have shown differences in effectiveness, such as a higher risk of *E. coli* growth with CHG and a higher risk of growth for *A. baumannii* and *Klebsiella* species with PI. However, not all these differences were statistically significant. Only one study, Salama *et al*^[33]^, showed that the growth of *S. epidermidis* was significantly higher with PI compared to CHG. This significance could be due to the larger sample size or the specific clinical context of Salama’s study, which might have amplified the effectiveness of CHG. Overall, these findings do provide some insight into the variability in the effectiveness of the antiseptics. However, only Salama *et al* shows a clear benefit of CHG over PI, and therefore the evidence does not consistently favor one antiseptic over the other to prevent the growth of these specific organisms. Sensitivity analyses were used to examine the possible effects of study quality, especially for results that had borderline statistical significance. We are certain that bias had little effect on the overall conclusions of this meta-analysis because the majority of the included studies were judged to have a low risk of bias.

Although more than 10 studies were included, formal tests for publication bias were not performed. The possibility of publication bias cannot be excluded.

### Clinical implications

In our meta-analysis, CHG has demonstrated significant clinical benefits as an antiseptic in its ability to reduce SSIs and lower bacterial colony-forming units (CFU). CHG is well tolerated by most patients; however, some studies have reported side effects when applied in higher concentrations of up to 4% and documented complete desquamation of the vagina^[39]^. This highlights the importance and need for further research regarding the ideal concentration of CHG to be used for preoperative skin antisepsis, such that it minimizes adverse reactions without compromising its bactericidal effects. An RCT comparing the bactericidal effects of CHG and PI reported that there was mild itching and burning, which was very rare, in women who received 1% CHG as the antiseptic^[40,41]^. Nonetheless, these effects are comparable to those seen with PI. Despite these rare occurrences, 0.05% CHG has shown very low toxicity and has been proven to be safe for wound healing and granulation tissue. There is also a low chance of adverse reaction with concentrations of 0.05–0.1%^[42]^. The ACOG considers up to 4% of CHG to be safe for use in gynecologic surgeries^[9]^. Another study also reported that preoperative skin preparation with CHG concentrations of 2–4% is safe and effective in preventing SSIs^[41]^. CHG is the antiseptic of choice, especially in patients who have iodine allergies^[9]^.

The economic data of the meta-analysis from the subgroup of the studies also showed significant differences in the costs for PI and CHG, and isopropyl alcohol. It is known that SSIs can prolong the time of a patient who stays in the hospital and hence can increase the cost of healthcare^[1]^. The findings of this research show that, although the costs of acquiring PI are comparatively lower than CHG, due to its initial cost advantage, which was initially considered to be more economical, numerous studies have revealed that CHG is more effective in practice, especially in the reduction of SSIs, which in turn leads to lower mean total healthcare costs.

For instance, Culligan *et al*^[26]^ and Hill *et al*^[27]^ noted that postoperative infection rates that are associated with longer stays in hospital, greater length of treatment, and the utilization of more healthcare resources were found to be significantly reduced by CHG. This was supported by Salama *et al*^[33]^, who showed hospital readmission was significant in the PI group, and Kesani *et al*^[28]^, who demonstrated that hospitals adopting CHG had reduced readmissions due to SSIs, hence reducing the long-term cost in contrast to the higher infection rate of the PI group. Likewise, our forest plot also showed that hospital readmission was significantly seen in PI patients.

In the subgroup analysis, when the population of different income statuses was seen, Albezrah *et al*^[25]^ and Lakhi *et al*^[29]^ showed that the risk of SSI was significant in the population of high-income countries in the PI group. Whereas in upper and lower-middle-income countries, Kesani *et al*^[28]^ and Salama *et al*^[33]^ showed that SSIs were significant in the PI group. In conclusion, our forest plot also showed that SSIs are significant in PI irrespective of income status.

This cost-benefit relationship between the antiseptics underscores the importance of adopting more effective antiseptic protocols to reduce postoperative complications and healthcare costs in the long term.

### Guidelines

The present guidelines on the prevention of SSIs have been enriched by this meta-analysis, in particular, the comparison of antiseptic agents such as CHG and PI. Medications that kill the microbes should be employed as recommended by the relevant authoritative groups, such as the Centers for Disease Control and Prevention (CDC) and the World Health Organization (WHO). However, the use of CHG and PI have differed mainly because of the differences in the findings of the studies conducted.

Two of the studies in this meta-analysis, Culligan *et al*^[26]^ and Kesani *et al*^[28]^ show that CHG reduces SSIs more compared to PI when used in conjunction with isopropyl alcohol. Overall, our findings endorse the utilization of CHG in surgical preparation, especially in high-risk surgeries, and correlate with the previous studies. As previously discussed, Gezer *et al*^[21]^ and Lakhi *et al*^[29]^ emphasized the need to standardize the concentration of CHG and application time to enhance the efficacy of this emollient. Additionally, studies such as Hill *et al*^[27]^ and Rockefeller *et al*^[32]^ suggest that our findings may prompt revisions in global healthcare guidelines, especially in regions with high infection rates or high-risk surgeries.

There had been issues with the proper dose and duration of the use of antiseptics, especially for CHG and PI, which were inconsistent in previous guides^[5]^. However, through our meta-analysis, we have presented compelling data that CHG reduces patient morbidity and healthcare costs associated with SSIs. We have extended and amplified the prior guidelines^[4,5]^ by including this evidence; the above-said recommendation has been made clearer and more specific, particularly urging CHG with isopropyl alcohol as the ideal antiseptic capable of preventing SSI in most of the surgical scenarios.

### Strengths, limitations, and future implications

A key strength of our study is the inclusion of the AMSTAR 2 checklist for quality assessment, which enhances the methodological rigor and credibility of our systematic review. We also utilized the TITAN checklist to ensure clarity, transparency, and completeness in reporting, further strengthening the reliability of our methodological approach. Additionally, a robust and comprehensive literature search was conducted across six databases, including PubMed, Google Scholar, Cochrane Library, and China National Knowledge Infrastructure, ensuring broad coverage of relevant literature. By including only RCTs, we focused exclusively on high-quality evidence.

This meta-analysis incorporated twelve RCTs with a combined total of 4936 participants, providing a strong statistical foundation. Additionally, a wide range of secondary outcomes was assessed in addition to primary endpoints, allowing for a more comprehensive evaluation of the antiseptic interventions. Potential confounders – such as differences in socioeconomic status and the varying concentrations of CHG used – were addressed through subgroup analyses, and sensitivity analyses were performed for all outcomes exhibiting high heterogeneity. Notably, more than 75% of the outcomes included in the GRADE assessment were rated as “High” or “Moderate” quality, further strengthening the confidence in our findings.

Despite these strengths, some limitations exist. The exclusion of non-English language studies may have introduced language bias. Additionally, heterogeneity in antiseptic preparations across trials, including variations in CHG and PI concentration and the presence or absence of alcohol, may have influenced the outcomes. Furthermore, many included studies did not consistently account for underlying conditions such as diabetes, anemia, preeclampsia, and other comorbidities that may influence the risk of SSIs, potentially introducing residual confounding. Other aspects, such as the long-term cost-effectiveness, duration of follow-up, and the potential additive antiseptic effect of alcohol, remain underexplored and warrant investigation in future studies.

## Conclusion

Our analysis suggests that compared to PI, CHG is a more effective and equally safe preoperative vaginal antiseptic for preventing SSIs after obstetric and gynecological operations, though it may vary depending on institutional protocols, patient allergies, and specific surgical considerations. This meta-analysis calls for updated clinical guidelines and further investigation into long-term outcomes and the cost-effectiveness of this approach. Favoring CHG over PI for preoperative vaginal antisepsis in future practice guidelines may help reduce the healthcare burden and morbidity associated with SSIs.

## Data Availability

All data used in this study are from previously published sources. The extracted datasets are available from the corresponding author upon reasonable request.

## Research registration unique identifying number (UIN)

The protocol for this study is registered with PROSPERO (CRD42024566304) and no deviation from the registered protocol was made during the conduct of this review.
